# Immobilization of BMP-2 and VEGF within Multilayered Polydopamine-Coated Scaffolds and the Resulting Osteogenic and Angiogenic Synergy of Co-Cultured Human Mesenchymal Stem Cells and Human Endothelial Progenitor Cells

**DOI:** 10.3390/ijms21176418

**Published:** 2020-09-03

**Authors:** Maria Godoy-Gallardo, Núria Portolés-Gil, Ana M. López-Periago, Concepción Domingo, Leticia Hosta-Rigau

**Affiliations:** 1 Department of Health Technology, Centre for Nanomedicine and Theranostics, DTU Health Tech, Technical University of Denmark, Produktionstorvet, Building 423, 2800 Kgs. Lyngby, Denmark; mgodoy@uic.es; 2 Materials Science Institute of Barcelona (ICMAB-CSIC), Campus de la UAB s/n, 08193 Bellaterra, Spain; nuria.portoles.gil@gmail.com (N.P.-G.); amlopez@icmab.es (A.M.L.-P.); conchi@icmab.es (C.D.)

**Keywords:** angiogenesis, bone tissue engineering, co-delivery, growth factors, multilayers, osteogenesis, polydopamine, scaffolds

## Abstract

We have previously reported the fabrication of a polycaprolactone and hydroxyapatite composite scaffold incorporating growth factors to be used for bone regeneration. Two growth factors were incorporated employing a multilayered coating based on polydopamine (PDA). In particular, Bone morphogenetic protein-2 (BMP-2) was bound onto the inner PDA layer while vascular endothelial growth factor (VEGF) was immobilized onto the outer one. Herein, the in vitro release of both growth factors is evaluated. A fastest VEGF delivery followed by a slow and more sustained release of BMP-2 was demonstrated, thus fitting the needs for bone tissue engineering applications. Due to the relevance of the crosstalk between bone-promoting and vessel-forming cells during bone healing, the functionalized scaffolds are further assessed on a co-culture setup of human mesenchymal stem cells and human endothelial progenitor cells. Osteogenic and angiogenic gene expression analysis indicates a synergistic effect between the growth factor-loaded scaffolds and the co-culture conditions. Taken together, these results indicate that the developed scaffolds hold great potential as an efficient platform for bone-tissue applications.

## 1. Introduction

Bones are a rigid and complex form of connective tissue that is constantly created and renewed throughout life. However, despite this capacity for regeneration and self-healing, large bone defects resulting from infection, trauma or tumour resection often result in failure to heal [1,2]. While current therapeutic methods mainly rely on autologous bone grafting, transplanting bone from a different part of the body into the defect site has a range of disadvantages. Such limitations include immune reactions, risk of infection, pain or need for additional surgery [3,4,5]. Additionally, bone autografts also suffer from limited availability [6,7].

The field of bone tissue engineering (BTE) has emerged as a powerful alternative by providing three-dimensional (3D) biomaterial-based scaffolds for regenerative treatments [8]. The aim is to implant the scaffold at the defect site, recruiting host cells and guiding them towards osteogenic differentiation in situ, thus promoting regeneration of the bone [9,10].

Currently, materials employed for the fabrication of scaffolds have mainly relied on bioceramics and biodegradable polymers [11]. Bioceramics such as calcium phosphates have attracted much interest due to their chemical composition, which resembles the natural mineral phase of bones, making them biocompatible and osteoconductive [12]. However, bioceramics also display some drawbacks, mainly related to their brittleness. In contrast, scaffolds based on synthetic polymers exhibit mechanical properties matching those of natural bone tissue. Therefore, composite scaffolds of bioceramics and polymers have been proposed [13,14]. One of the most popular consists of hydroxyapatite (HA) nanoparticles (HA-NPs) dispersed within a poly(ε-caprolactone) (PCL) matrix [13,14]. We have previously reported the fabrication of PCL/HA-based scaffolds displaying a highly interconnected network of pores with a bimodal pore size distribution [15]. Such an architecture mimics the extracellular matrix of natural bone tissue. While the macropores of the ECM promote cell growth, proliferation and migration, the microporous network grants diffusion of the necessary oxygen, nutrients and waste. However, the hydrophobicity of PCL still renders scaffolds with insufficient cell adhesion and proliferation, while HA alone displays limited osteoinductive ability [12]. Thus, we functionalized the PCL/HA-based scaffolds with a combination of osteoinductive and angiogenic growth factors (GFs) [16]. Co-delivery of both types of GFs is a powerful approach to promote vascularized bone regeneration [10]. Since bone is a highly mineralized and vascularized tissue, insufficient angiogenesis results in poor and unsustainable bone formation [17]. We chose to immobilize bone morphogenetic protein-2 (BMP-2) and vascular endothelial growth factor (VEGF), since such a combination has demonstrated the ability to promote augmented bone regeneration [18]. While BMP-2 is a potent osteogenic GF already approved by the FDA [19], VEGF plays an important role in the formation of blood vessels. Furthermore, VEGF has the ability to promote recruitment of endothelial progenitor cells (EPCs) and their differentiation into endothelial cells [19] while also stimulating the recruitment of osteoblastic cells [10]. As a strategy to immobilize GFs onto the scaffolds, we employed self-polymerization of dopamine (DA) into polydopamine (PDA). This in situ self-polymerization process results in a PDA coating which can adhere into virtually any substrate independently of the constituting material, size or shape [12,20,21,22,23]. This results in an efficient strategy to incorporate biomolecules onto different substrates without the need for surface pretreatment [12,24]. Additionally, PDA coatings have also demonstrated the ability to promote cell adhesion and proliferation of various cell lines including osteoblasts [25], a feature that can further improve the osteointegration of polymer-based scaffolds. Thus, we prepared PCL/HA composite scaffolds with a bimodal distribution of pore sizes and incorporated both BMP2 and VEGF by means of a PDA coating.

**Scheme 1:**
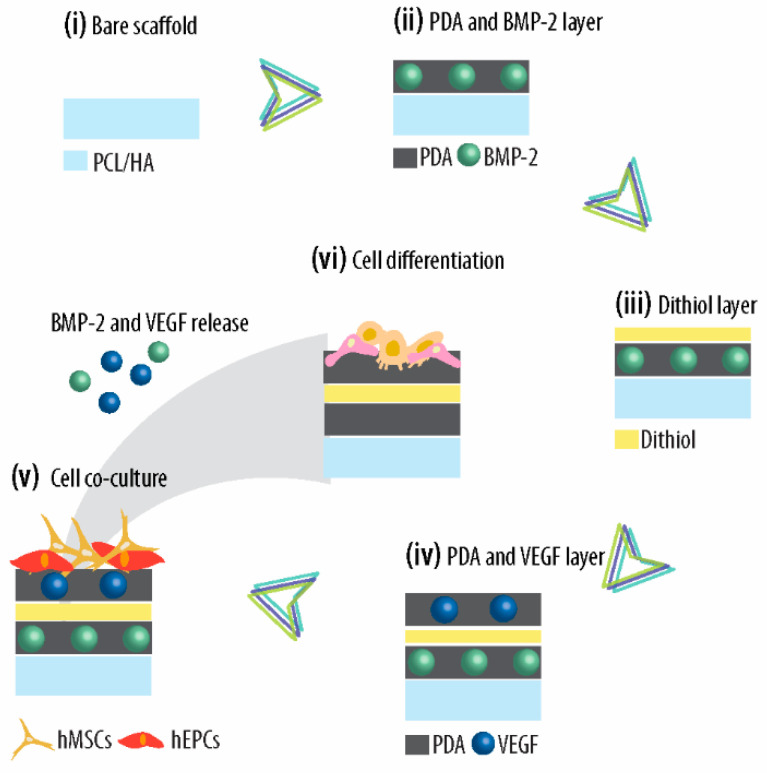
(**i**) Poly(ε-caprolactone) (PCL)/hydroxyapatite (HA)-based scaffolds are first coated by a polydopamine (PDA) layer incorporating the growth factor bone morphogenetic protein-2 (BMP)-2 (**ii**). Next, an interconnecting dithiol layer is deposited (**iii**) to allow for coating with a second PDA layer containing the vascular endothelial growth factor (VEGF) growth factor (**iv**). The coated scaffolds are evaluated by co-culturing human mesenchymal stem cells (hMSCs) and human endothelial progenitor cells (hEPCs) (**v**) and by assessing their differentiation into bone-forming and vessel-forming cells by gene expression (**vi**).

While, in our previous work, we identified the conditions promoting the highest incorporation of GFs and osteogenic differentiation of human mesenchymal stem cells (hMSCs) [16], herein, we further characterize the optimal assembly in terms of osteogenic but also angiogenic potential. Specifically, we fabricate highly interconnected porous PCL/HA composite scaffolds with a bimodal pore size distribution which are subsequently coated by two independent PDA layers loaded with the two GFs (Scheme 1). Since the secretion of BMP-2 occurs during the entire healing process [19], BMP-2 is loaded in the first and more hindered PDA layer. In contrast, VEGF expression takes place mainly at the early healing stage [26]. Therefore, VEGF is immobilized within the second and more exposed layer. Following assembly and characterization of the surface coatings, we evaluate the potential of the scaffolds for BTE applications in terms of the angiogenic and osteogenic gene expression in a co-culture system of hMSCs and human EPCs (hEPCs). This is an important aspect since, the interplay between vessel-forming endothelial cells and bone-forming cells is a crucial aspect in bone healing, involving several and interconnected steps overlapping in time [27].

## 2. Results and Discussion

### 2.1. Fabrication and Characterization of PCL/HA-Based Scaffolds

Many studies have recently focused on the fabrication of composite scaffolds of HA-NPs and PCL [7,28]. Although 70% of the mineral phase of bone is constituted by HA due to the brittleness of bioceramics, HA is usually employed in combination with synthetic polymers. In particular, HA is employed in combination with PCL [7,28], which is an FDA-approved polyester for craniofacial indications [29]. Furthermore, HA/PCL-based scaffolds have been recently prepared displaying a bimodal pore size distribution of highly interconnected pores [15]. This is an important feature since, within the extracellular matrix of natural bone tissue, few hundred-sized macropores allow for 3D cell adhesion, proliferation and migration. In contrast, micron-sized pores (usually below 100 µm) are essential for the transport of nutrients and waste [30,31].

**Figure 1:**
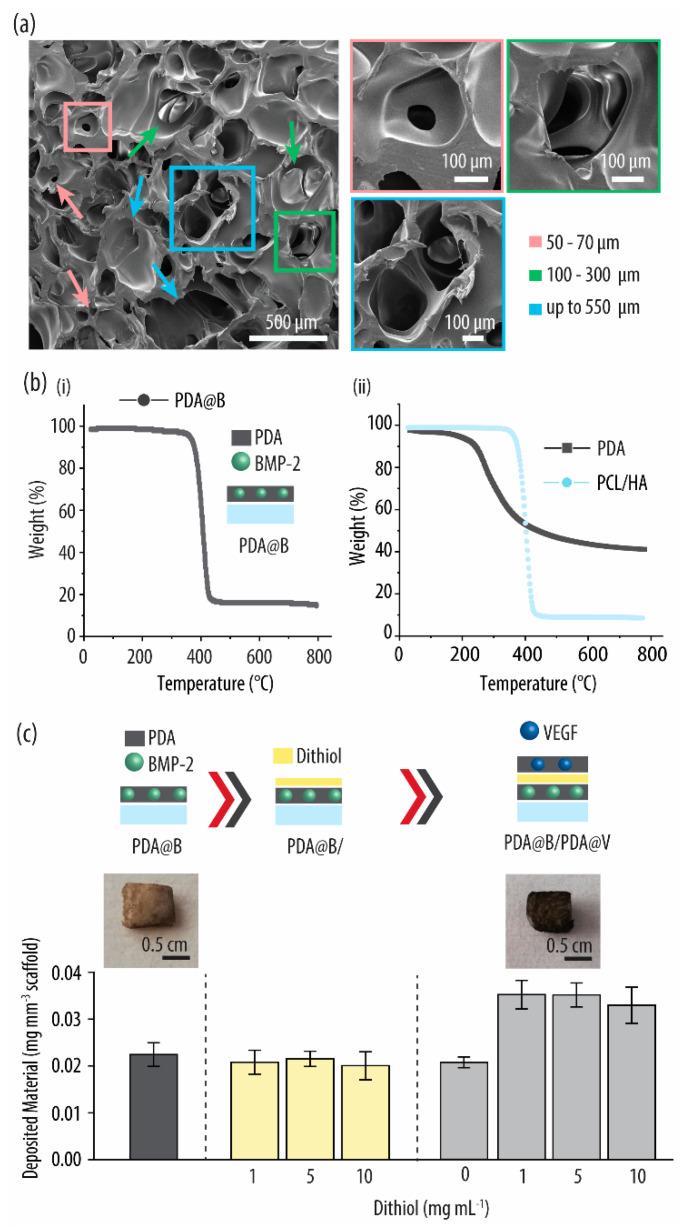
(**a**) Representative scanning electron microscopy images of a cross section of the PCL/HA composite scaffolds: while the pink arrows indicate pores of a size of 50–70 µm, the green and blue arrows indicate macropores of 100–300 µm and extra-large pores up to 550 µm, respectively. (**b**) Thermal degradation of the PCL/HA-based scaffold coated by a polydopamine (PDA) layer incorporating BMP-2 (PDA@B) (i) and the corresponding controls of bare PCL/HA scaffolds and PDA only. (**c**) Amount of deposited material onto the scaffolds as assessed by thermogravimetric analysis after each deposition step depending on the concentration of dithiol employed: PCL/HA scaffolds are first coated by a PDA@B layer and incubated in a dithiol solution at three different concentrations. Finally, a PDA layer containing VEGF (PDA@V) is adsorbed on a second deposition step (PDA@B/PDA@V).

Thus, following a previously reported protocol [15,31], PCL/HA-based scaffolds were prepared employing a super critical (sc) CO_2_ foaming procedure in which a two-step depressurization process was applied. Figure 1a shows scanning electron microscopy (SEM) images of the cross section of a scaffold sample exhibiting a double scale of pore sizes. Micro-sized (from 50 to 70 µm), macro-sized (from 100 to 300 µm) and extra-large (up to 550 µm) pores can be observed, all of them important to enhancing angiogenesis and to strengthening bone regeneration [5]. Such a cross-sectional image also shows the high interconnectivity among the different pores. While herein, only the effect of pore size and interconnectivity have been investigated, it is also worth mentioning that the pore geometry (i.e., shape) is also of relevance in BTE. For example, the curvature of the radius, convexity as well as the presence and size of the angles can be related to tissue growth [32].

The porosity of the scaffolds was also calculated, and a porosity value of 74 ± 5% was obtained. Such a porosity value is well within the porosity range of natural bone tissue [33] and is particularly well suited for allowing cell penetration and efficient vascularization of the developing tissue [34].

### 2.2. Surface Functionalization

Administering a combination of VEGF and BMP-2 can lead to enhanced bone regeneration as compared to delivering BMP-2 alone [10,35]. However, both GFs display a very short half-life in vivo. Additionally, due to the lack of adequate sustained delivery systems, at present, supraphysiological levels of both GFs have to be used in the formulations [10,19]. As an example, current clinical approaches employing collagen scaffolds for BMP-2 delivery require BMP-2 quantities that are six orders of magnitude higher than the amount of BMP-2 present in a bone defect that is healing naturally [36,37]. Obviously, such an approach results in high costs but also safety concerns due to undesirable side effects [38]. Thus, there is an increasing need to develop sophisticated scaffolds with the ability to deliver several GFs with controlled delivery kinetics. Herein, we fabricate multilayered PDA-coated scaffolds and entrap both BMP-2 and VEGF within different/separated PDA layers. As a first step, BMP-2 was immobilized by incubating the PCL/HA scaffolds in a DA solution containing BMP-2 at a concentration of 1 µg mL^−1^. The amount immobilized within the scaffold was assessed by quantifying the BMP-2 remaining in the DA solution after the incubation time. The results showed that 759 ± 60 ng of BMP-2 per 50 mg of scaffold had been efficiently immobilized, which corresponds to approximately 80.1% of the total added BMP-2. This is an important fact since achieving a high-binding efficiency is highly desirable, crucial due to their extremely high costs (i.e., usually over $5.000 mg^−1^) [37].

The amount of PDA deposited within the first layer was evaluated by thermogravimetric analysis (TGA). The scaffolds were heated from 30 to 800 °C at a rate of 10 °C min^−1^, and the mass loss as a function of temperature was monitored. Figure 1b(i) shows thermal degradation of the coated scaffold beginning at approximately 200 °C and leaving approximately 18 wt % residue upon reaching 450 °C. The control experiments show how, upon reaching a temperature of approximately 700 °C, approximately 40 wt % residue containing both PDA and char remained in the sample (Figure 1b(ii)). For uncoated PCL/HA scaffolds, the control experiments show thermal degradation starting at approximately 400 °C, leaving approximately 10 wt % residue upon reaching a temperature of 450 °C. Since previous studies have shown that HA-NPs alone result in a residue at 650 °C close to its nominal amount [31], it can be concluded that this 10 wt % residue corresponds to HA. Additionally, since PCL completely degrades upon reaching 400 °C, it is suggested that approximately 18 wt % residue left from the TGA data of the PDA and BMP-2-coated scaffolds (PDA@B) corresponds to both PDA and HA.

**Figure A1:**
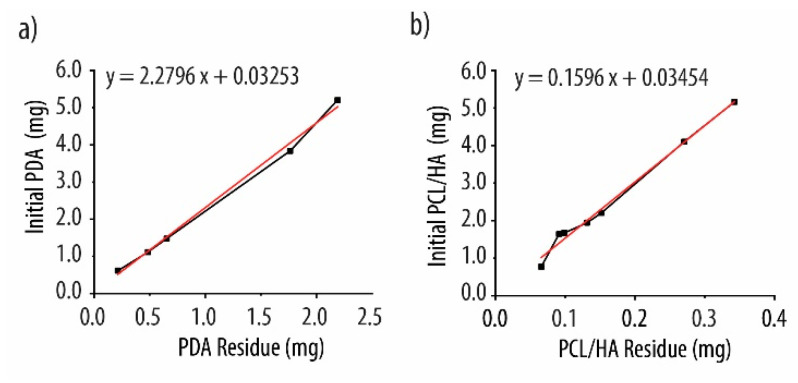
Plots of different initial weights of samples against the residual weights at 800 °C for both PDA (**a**) and PCL/HA scaffolds (**b**).

In order to determine the amount of PDA deposited (*M*_PDA_) onto the scaffolds, several TGA curves of different amounts of starting PDA powder and PCL/HA scaffolds were studied. Initial weights of the samples vs. the residual weights at 800 °C were plotted (Figure A1), and subsequently, *M*_PDA_ was calculated with the following equations:

(1) $$M_{\mathrm{PCL}/\mathrm{HA}}=0.1596*m_{\mathrm{PCL}/\mathrm{HA}}+0.03454$$

(2) $$m_{\mathrm{PCL}/\mathrm{HA}-\mathrm{PDA}}=m_{\mathrm{PDA}}+m_{\mathrm{PCL}/\mathrm{HA}}$$

(3) $$M_{\mathrm{PDA}}=2.2796*m_{\mathrm{PDA}}+0.03253$$

In the equation, *m*_PDA_ corresponds to the residual weight for PDA, *m*_PCL/HA_ corresponds to the residual weight for PCL/HA and *m*_PCL/HA_PDA_ corresponds to the residual weights for PDA-coated scaffolds, as shown by the TGA curves at 800 °C. *M*_PDA_ and *M*_PCL/HA_ indicate the weights of PDA and the uncoated scaffolds, respectively, before starting the TGA experiment. This results in approximately 0.022 mg of PDA per mm^3^ of scaffold.

Next, in order to adsorb a second PDA layer, which will allow for independently loading different GFs in a controlled manner, a connecting linker needs to be deposited. We chose 1,9-nonanedithiol, a dithiol linker which can react with the first PDA layer by Schiff base reaction and Michael addition while providing additional thiol groups that will allow to adsorb an additional PDA layer [39]. Thus, the PDA-coated scaffolds were incubated in dithiol solutions at concentrations of 1, 5 and 10 mg mL^−1^, respectively, and the amount of PDA deposited in this second step was quantified by TGA. Figure 1c shows the PDA@B adsorbed on a first deposition step (dark grey bars). Upon incubating the PDA@B scaffolds in the three different dithiol solutions, no increase in the amount of deposited material was observed (yellow bars). Next, the amount of PDA-containing VEGF (PDA@V) on a second deposition step depending on the dithiol concentration was evaluated. The scaffolds were incubated in a DA solution containing VEGF at a concentration of 0.5 µg mL^−1^. An approximately 1.5-fold increase in the amount of PDA@V deposited in this second step can be observed for the three different dithiol concentrations (light grey bars). Importantly, in the absence of dithiol, no increase in the amount of deposited material could be detected, thus highlighting the importance of incorporating a “connecting” linker. Since no differences could be detected depending on the dithiol concentration, further experiments were conducted employing the lowest concentration (i.e., 1 mg mL^−1^). The successful deposition of PDA onto the scaffolds was also corroborated by the colour change from light brown to black that is characteristic of PDA. To evaluate the incorporation efficiency, the amount of unincorporated VEGF was quantified by collecting the supernatant after completing the incubation time. The results showed that 329 ± 43 ng of VEGF per 50 mg of scaffold was immobilized, corresponding to 61.8% of the total added VEGF. As previously mentioned, such a high entrapment efficiency is highly desirable in this context due to the high cost of GFs.

**Figure A2:**
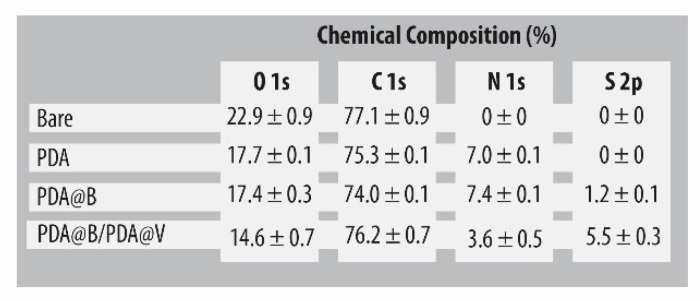
Surface chemical composition for the three different scaffolds as assessed by X-ray photoelectron spectroscopy. Bare scaffolds have been coated with polydopamine (PDA) only, PDA entrapping BMP-2 (PDA@B) and PDA entrapping both BMP-2 and VEGF growth factors (PDA@B/PDA@V).

To draw a stronger conclusion over the incorporation of BMP-2 and VEGF onto the scaffolds after the different deposition steps, the coated scaffolds were analysed by X-ray photoelectron spectroscopy (XPS). Figure A2 shows the quantification of the atomic constitution. As expected, no N 1s content was detected for the uncoated PCL/HA scaffolds while PDA coating resulted in 7.0 ± 0.1% N 1s content. Only the scaffolds incorporating the GFs showed S 2p content, specifically, 1.2 ± 0.1% S2p content for scaffolds incorporating BMP-2 only (PDA@B) and 5.5 ± 0.3% S2p content for scaffolds incorporating both BMP-2 and VEGF (PDA@B/PDA@V). The S 2p content can unequivocally be attributed to the sulphur of the amino acids present in BMP-2 for PDA@B and to the sulphur present in both GFs and also in the dithiol linker for PDA@B/PDA@V.

### 2.3. In Vitro Release Kinetics of BMP-2 and VEGF

**Figure 2:**
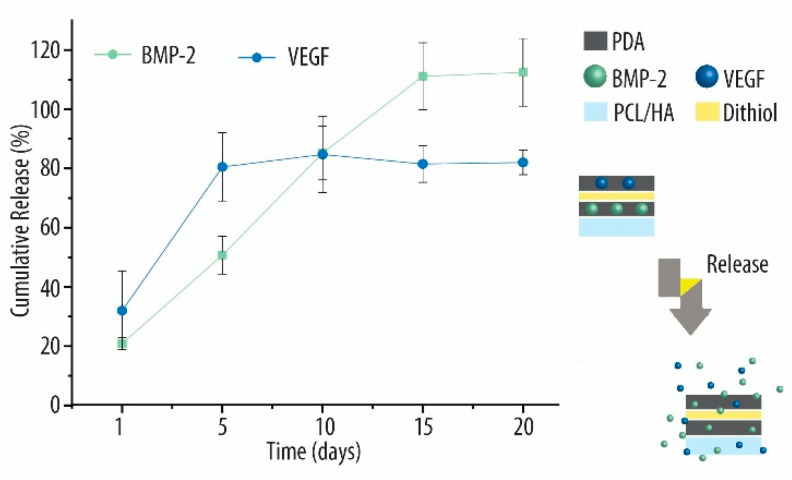
Release profiles of BMP-2 and VEGF from the scaffolds: PCL/HA-composite scaffolds have been coated with polydopamine (PDA) entrapping BMP-2 and VEGF growth factors within two different layers (PDA@B/PDA@V). A dithiol linker has been incorporated to allow for the deposition of a second PDA layer.

The release profiles of BMP-2 and VEGF from the PDA@B/PDA@V-coated scaffolds were identified by enzyme-linked immunosorbent assay (ELISA). Figure 2 shows different release behaviours for BMP-2 and VEGF. In particular, BMP-2 exhibited a burst release on day 1 (approximately 20% release) followed by approximately 7–8% release per day up to day 5. Next, a substantially longer release of approximately 4% per day until day 20 was detected. In contrast, VEGF exhibited a substantially higher extent of burst release on the first 5 days (approximately 80% of VEGF), thus mimicking VEGF expression during the early bone healing stage. Complete VEGF release was achieved on day 7. Importantly, approximately 100% and 80% of all immobilized BMP-2 and VEGF, respectively, were released at the end of the 20 days of incubation. Hence, it can be hypothesized that the fastest VEGF release is a result of it being incorporated within the second and most exposed PDA layer. In contrast, the slower and sustained release of BMP-2 is a consequence of it being incorporated into the inner PDA layer. Such release kinetics, where VEGF is fast released while the BMP-2 release is more sustained and prolonged, is a desired feature for scaffolds to be employed in BTE applications. Within our body, secretion of BMP-2 occurs during the entire process of bone healing [19] while VEGF expression takes place mainly at an early stage [26]. It is also worth noticing that, while PDA coatings have been previously used to immobilize GFs [12,40], to the best of our knowledge, this is the first time that PDA coatings are used to achieve control over biomolecule dosing. This is accomplished in this work by creating two independent PDA layers.

### 2.4. Proliferation and Adhesion of hMSCs and hEPCs

**Figure 3:**
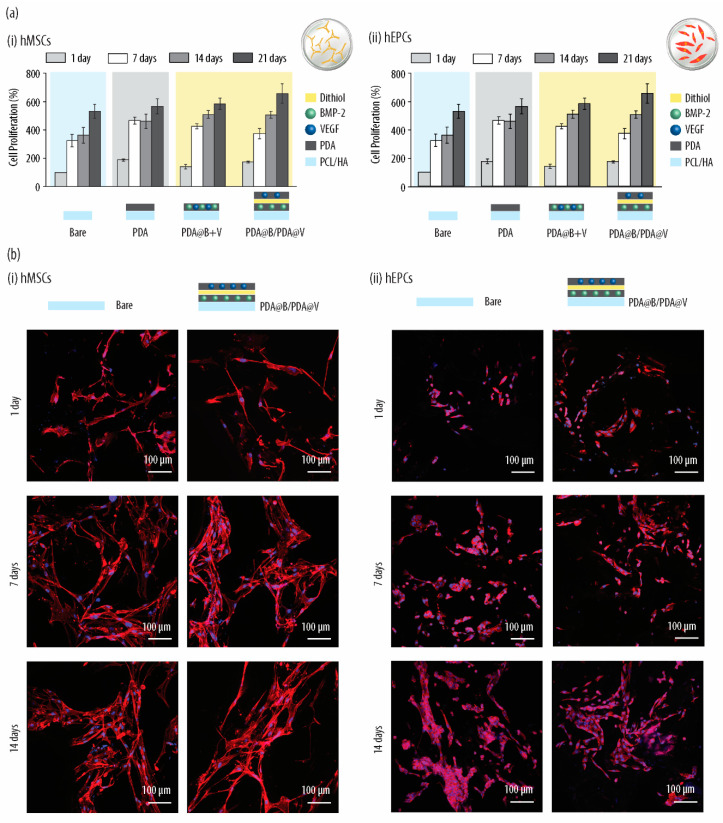
(**a**) Proliferation of human mesenchymal stem cells (hMSCs) (**i**) and human endothelial progenitor cells (hEPCs) (**ii**) onto PCL/HA scaffolds (bare), surface coated with polydopamine only (PDA), entrapping both BMP-2 (B) and VEGF (V) growth factors within the same (PDA@B+V) or in two different (PDA@B/PDA@V) PDA layers: A dithiol linker has been added to allow for the deposition of a second PDA layer. Both hMSCs and hEPCs have been cultured for 1, 7, 14 and 21 days. (**b**) Merged confocal laser scanning microscopy images of hMSCs (**i**) and hEPCs (**ii**) cultured onto bare and PDA@B/PDA@V-coated scaffolds for several time intervals (i.e., 1, 7 and 14 days): The actin filaments have been stained with palloidin-TRITC (red fluorescence signal), and the nuclei have been stained with 4′,6-diamidino-2-phenylindole (DAPI) (blue fluorescence signal).

**Figure A3:**
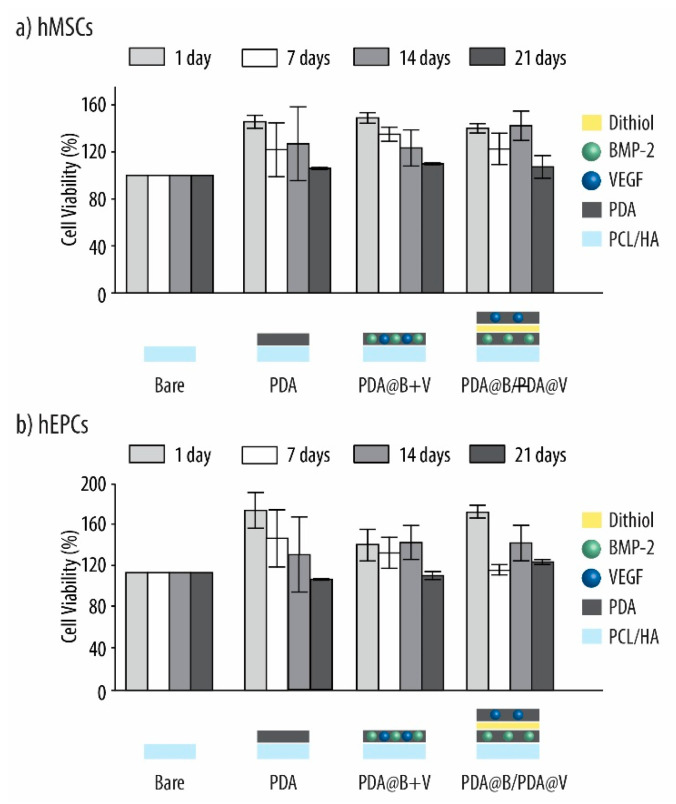
Cell viability of human mesenchymal stem cells (hMSCs) (**a**) and human endothelial progenitor cells (hEPCs) (**b**) onto PCL/HA scaffolds (bare), surface coated with polydopamine only (PDA), entrapping both BMP-2 (B) and VEGF (V) growth factors within the same (PDA@B+V) or in two different (PDA@B/PDA@V) PDA layers.

To achieve positive therapeutic outcomes in bone regeneration, developing angiogenesis and a functional microvasculature within the bone tissue constructs are a crucial aspect [17]. Bone healing is a complex process, involving not only bone forming cells but also endothelial cells. As such, the implanted scaffolds should exhibit angiogenic properties also during the first stages of bone regeneration. Thus, the ability of the developed surface coating (PDA@B/PDA@V) to modulate the proliferation and cell adhesion of both hMSCs and hEPCs was investigated and compared to the uncoated PCL/HA composite scaffolds. Additionally, scaffolds where BMP-2 and VEGF had been entrapped within a single PDA layer (PDA@B+V) were also investigated. Prior to assessing the proliferation and cell adhesion of both cell lines, cell viability assays were conducted, confirming the biocompatibility of the scaffolds (Figure A3). Figure 3a shows how both hMSCs and hEPCs progressively increased their numbers in all types of scaffolds. However, for both types of cells, slightly increased numbers of cells were observed for the coated scaffolds (i.e., PDA, PDA@B+V and PDA@B/PDA@V) as compared to the uncoated ones. The highest proliferation was observed for the coated scaffolds incorporating the GFs in two separate PDA layers (i.e., PDA@B/PDA@V). Specifically, for hMSCs seeded onto PDA@B/PDA@V-coated scaffolds, approximately 2-, 1.1-, 1.3- and 1.2-fold increases in cell proliferation were observed after 1, 7, 14 and 21 days, respectively, as compared to the bare ones. For hEPCs, the results were very similar, and for PDA@B/PDA@V-coated scaffolds, approximately 2-, 1.3-, 1.4- and 1.2-fold increases in cell proliferation could be observed after 1, 7, 14 and 21 days, respectively, as compared to the uncoated ones. Next, the morphologies of both cell lines seeded onto the different scaffolds were evaluated after 1, 7 and 14 days of culturing. For that, the acting filaments and nuclei of the cells were stained and visualized by confocal laser scanning microscopy (CLSM). Figure 3b shows how, while similar adhesion of hMSCs was observed for both the bare and coated (PDA@B/PDA@V) scaffolds for all studied time intervals, the results were different for hEPCs. hMSCs displayed a spread morphology with a well-defined cytoskeleton independent of the presence of a surface coating. In contrast, after 1 day of cell culturing, hEPCs showed lower cell adhesion for bare substrates as compared to the coated ones. However, similar cell adhesion was observed following 7 and 14 days of cell culturing. Thus, to sum up, enhanced proliferation of both cell types was observed for the coated scaffolds, which can be attributed to the presence of both PDA layers and to the GFs. Both cell types were also able to adhere onto the scaffolds independently of the coating. This is not surprising since PCL/HA scaffolds have been previously reported to support cell adhesion and proliferation [31,41].

### 2.5. Gene Expression

Within this study, the effects of the surface coating on the in vitro expression of osteogenic differentiation markers (runt-related transcription factor 2 (RUNX-2), alkaline phosphatase (ALP) and osteocalcin (OCN)) and angiogenic markers (vascular endothelial growth factor A (VEGF-A), kinase insert domain receptor (VEGF-R2) and endothelin 1 (EDH-1)) were investigated in both hMSCs and hEPCs.

#### 2.5.1. Osteogenic Gene Expression

**Figure 4:**
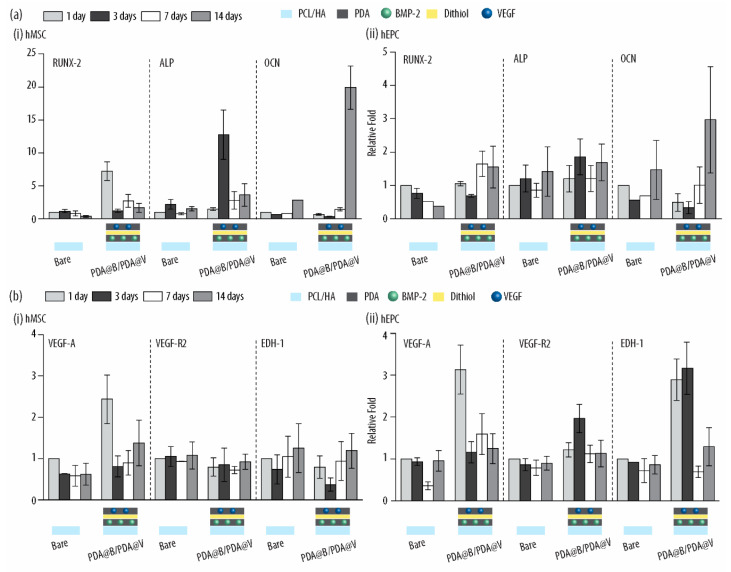
Real-Time quantitative reverse transcription polymerase chain reaction (qRT-PCR) analysis to assess the expression of osteogenic (**a**) and angiogenic (**b**) markers in human mesenchymal stem cells (hMSCs) (**i**) and human endothelial progenitor cells (hEPCs) (**ii**): Both cell lines were cultured onto PCL/HA scaffolds (bare), surface coated with polydopamine only (PDA), entrapping both BMP-2 (B) and VEGF (V) growth factors within two different PDA layers (PDA@B/PDA@V). A dithiol linker has been added to allow for the deposition of two PDA layers. Both hMSCs and hEPCs have been cultured for 1, 7, 14 and 21 days.

Osteogenic differentiation is a multi-step process controlled by complex molecular mechanisms and factors. The production or synthesis of RUNX-2, ALP and OCN occurs at different cell phases and is closely associated with osteogenic differentiation and calcification during the bone forming process [42]. For example, while RUNX-2 and ALP are observed at an early stage of the differentiation process, OCN expression takes place at a middle/late stage [12]. Thus, in a next step of the characterization of the PDA@B/PDA@V-coated scaffolds, the expressions of RUNX-2, ALP and OCN by quantitative real-time reverse transcription-polymerase chain reaction (qRT-PCR) after 1, 3, 7 and 14 days of culture were investigated. Figure 4a(i) shows how the incorporation of the PDA@B/PDA@V coating onto the scaffolds results in a very pronounced increase on the expression levels of the three osteogenic markers for hMSCs. In particular, approximately 7.5-, 5.0- and 6.6-fold increases for RUNX-2, ALP and OCN expression levels, respectively, were observed for the coated scaffolds as compared to the bare ones. These results indicate that the PDA@B/PDA@V coating has the ability to enhance the osteogenic activity of hMSCs. Interestingly, for hEPCs, upregulation osteogenic genes for PDA@B/PDA@V-coated scaffolds was also observed for ALP and, more pronouncedly, for OCN (i.e., approximately 2.0-fold increase). This result contributes to the increasing evidence of the connection between bone and vasculature [43].

#### 2.5.2. Angiogenic Gene Expression

VEGF plays an essential role in angiogenesis by attracting both osteoprogenitor and endothelial cells. By secreting trophic factors, the different cell types create a unique environment that mutually enhances their proliferation and survival [42,44]. Thus, the angiogenic potential of the VEGF-loaded PDA@B/PDA@V surface coating in both hMSCs and hEPCs was evaluated. VEGF-A, VEGF-R2 and EDH-1 were selected as angiogenic markers since these three GFs display crucial roles in the process of angiogenesis. While VEGF-A regulates the recruitment of hEPCs enhancing their proliferation and differentiation, VEGF-R2 is the main receptor mediating the angiogenic effects of VEGF-A [45]. EDH-1 has a dual function and regulates angiogenesis but also enhances bone formation [46]. Figure 4b shows how VEGF-A was upregulated in both cell lines for the PDA@B/PDA@V surface coating. This is not surprising since hMSCs also secrete VEGF to increase the proliferation of endothelial cells and thus promote vessel growth [47]. Such an upregulation was more pronounced for hEPCs (approximately 3.2-fold) as compared to hMSCs (approximately 2.5-fold). VEGF-A followed the same expression pattern in both cell lines, peaking at day 1 followed by a sharp decrease at day 3. Upregulation at day 1 has been previously reported, since VEGF-A is an early angiogenic marker [48]. In contrast, VEGF-R2 and EDH-1 were only upregulated in hEPCs for the PDA@B/PDA@V-coated scaffolds. The expression of both markers peaked at a later stage (i.e., at day 3 showing a sharp decrease for longer time intervals), and approximately 2.3- and 2.6-fold increases in the expression of VEGF-R2 and EDH-1, respectively, were observed for the coated scaffolds.

### 2.6. Optimization of hMSCs and hEPCs Co-Culture Ratio

**Figure 5:**
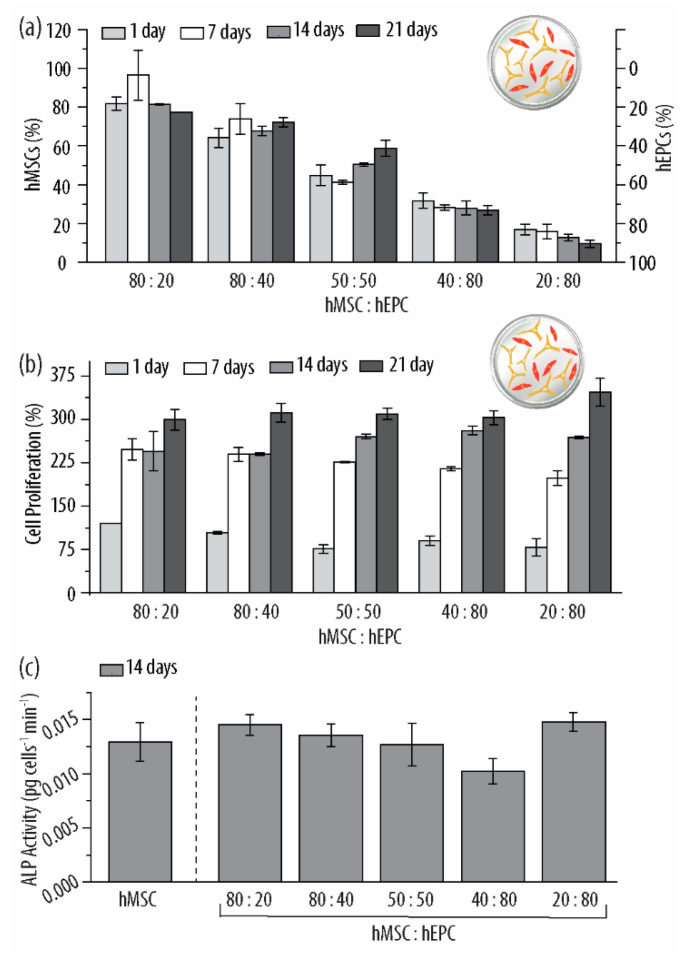
(**a**) Percentage of human mesenchymal stem cells (hMSCs) and human endothelial progenitor cells (hEPCs) for different seeding ratios after 1, 7, 14 and 21 days of co-culture; (**b**) proliferation of co-cultured hMSCs and hEPCs depending on the seeding ratio for different co-culturing time intervals; and (**c**) Alkaline phosphatase (ALP) activity readings of hMSCs and hEPCs co-cultured for 14 days at a 50:50 seeding ratio: The cells had been cultured onto PCL/HA scaffolds coated with polydopamine (PDA) layers entrapping both BMP-2 (B) and VEGF (V) growth in two different PDA layers (PDA@B/PDA@V). A dithiol connecting linker has been added to allow for the deposition of two PDA layers.

The interplay among vessel- and bone-forming cells is a crucial phenomenon that takes place during the early stages of bone healing. Thus, the scaffold materials to be used as bone implants should actively participate in the early stages of bone regeneration by stimulating a positive crosstalk among the different cells involved [27]. Several reports have already investigated the effects of different scaffolds on osteogenesis and angiogenesis in a co-culture setup [49]. For example, polysaccharide-based scaffolds enhanced the osteogenic potential of hMSCs when they were co-cultured with endothelial cells [50]. Scaffolds composed of silk fibroin have also showed the ability to promote the growth of endothelial cells in co-culture with hMSCs as opposed to monocultures [51]. Recently, angiogenic and osteogenic synergy of hMSCs and HUVEC was reported when co-cultured on a peptide amphiphilic nanomatrix functionalized by RGD [52]. Thus, the influence of PDA@B/PDA@V surface coating on the interaction of hMSCs and hEPCs was investigated. This was done by evaluating the angiogenic and osteogenic events in co-culture conditions. As a first step, the optimal ratio between the two cell lines was investigated. Figure 5a shows the percentage of the two cell types at different studied time intervals (i.e., 1, 7, 14 and 21 days) depending on the seeding ratio. Interestingly, the ratio between the two cell types upon seeding was maintained after the different time intervals for most of the studied conditions. As an example, when hMSCs and hEPCs were seeded at an 80:20 ratio, approximately 80% of the cells were identified as hMSCs following 1, 7, 14 and 21 days of cell culture. The results were slightly different for higher amounts of hEPCs. Specifically, upon seeding hMSCs and hEPCs at a 20:80 ratio, only approximately 15% and 10% of the cells were identified as hMSCs after 1 and 21 days of cell culture, respectively. The proliferation potential was found to be the same for all studied ratios and cell culture times (Figure 5b). Cell differentiation was also evaluated by measuring the ALP activity (Figure 5c). Assessment of ALP activity was chosen due to the central role of ALP in the initiation of the mineralization process by cell differentiation, displaying the highest activity during the pre-osteoblast differentiation stage [12]. ALP was evaluated following 14 days of cell culture since it has been previously demonstrated that the ALP activity of hMSCs cultured onto the developed substrates peaked at day 14 [16]. Interestingly, a similar ALP activity of approximately 15 × 10^−3^ pg cells^−1^ min^−1^ was observed for all studied ratios including the hMSC monoculture. Thus, a 50:50 ratio was chosen for the following experiments since such a ratio resulted in approximately the same percentage of both cell types at all studied time intervals. Furthermore, such a ratio also showed both high proliferation rates and ALP activity.

### 2.7. Cell Adhesion of Co-Cultured hMSCs and hEPCs

**Figure 6:**
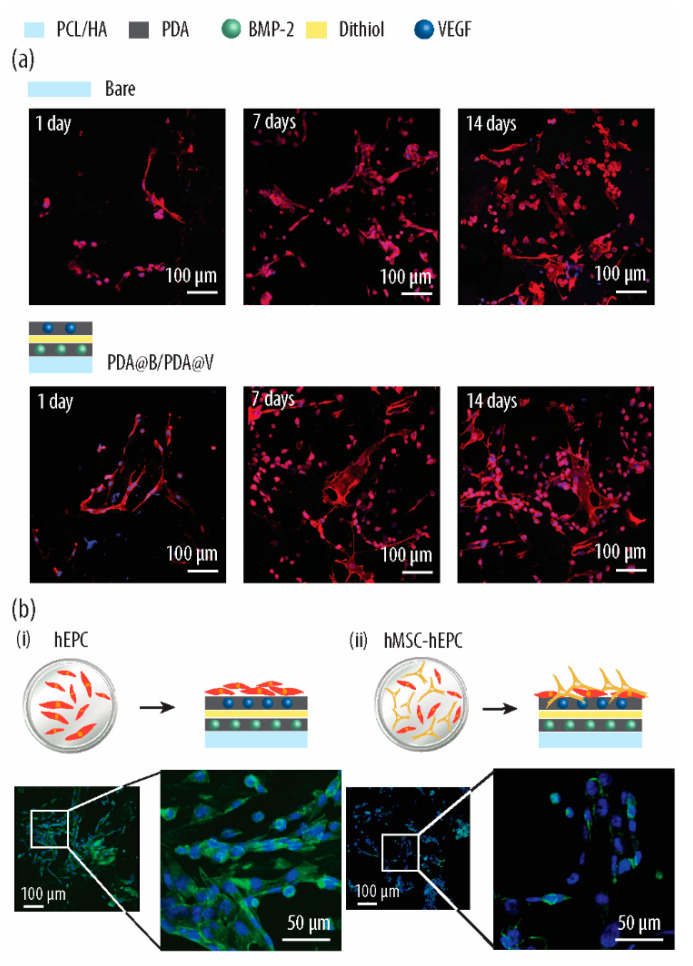
(**a**) Merged confocal laser scanning microscopy (CLSM) images of co-cultured human mesenchymal stem cells (hMSCs) and human endothelial progenitor cells (hEPCs) onto uncoated PCL/HA scaffolds (bare) or coated (PDA@B/PDA@V) scaffolds for 1, 7 and 14 days, respectively: the actin filaments have been stained with palloidin-TRITC (red fluorescence signal), and the nuclei have been stained with DAPI (blue fluorescence signal). (**b**) Merged CLSM images of monocultured hEPCs (**i**) and co-cultured hMSCs and hEPCs (**ii**) onto coated (PDA@B/PDA@V) scaffolds for 14 days: platelet endothelial cell adhesion molecule (PECAM-1) has been stained with Alexa Fluor 488 (green fluorescence signal), and the nuclei has been stained with DAPI (blue fluorescence signal). The PDA@B/PDA@V coating consists of two polydopamine (PDA) layers connected by a dithiol linker and entrapping BMP-2 (B) and VEGF (V) growth factors within the first and second PDA layers, respectively.

The morphologies of the two cell types in co-culture conditions were evaluated by CLSM. Figure 6a shows CLSM images of hMSCs and hEPCs seeded at a 50:50 ratio and co-cultured onto bare or PDA@B/PDA@V-coated substrates for 1, 7 and 14 days. The actin filaments and nuclei of the cells were stained. As expected, the cells progressively increased their number for both types of scaffolds. However, the cells showed enhanced cell adhesion for the coated scaffolds, as shown by their spread morphology. This fact can be attributed to the presence of both PDA and GFs. To discern among hEPCs and hMSCs in co-culture conditions, the cells were stained with endothelial-specific platelet endothelial cell adhesion molecule (PECAM-1) and were compared to monocultured hEPCs. Figure 6b shows a number of PECAM-1-stained cells in co-culture conditions, thus confirming the presence of the two types of cells.

### 2.8. Osteogenesis of hMSCs in Co-Culture Conditions

**Figure 7:**
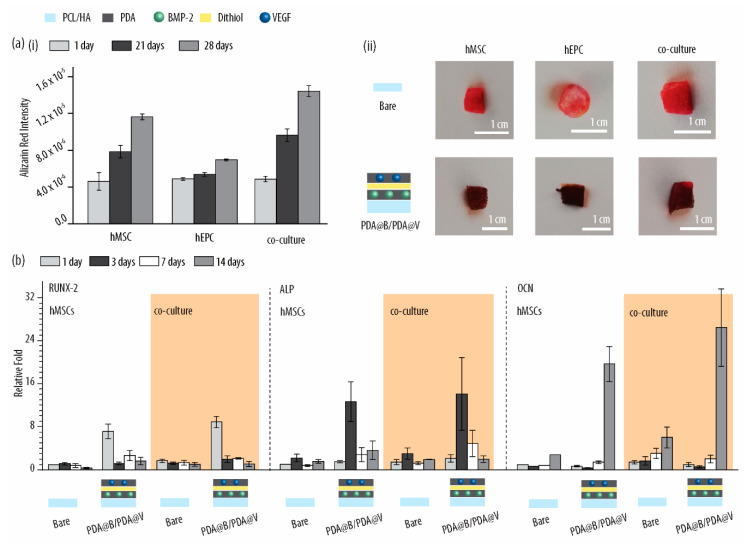
(**a**) (**i**) Alizarin red intensity of human mesenchymal stem cells (hMSCs), human endothelial progenitor cells (hEPCs) and the co-culture of both cells at a 50:50 seeding ratio cultured onto surface coated scaffolds; (**ii**) macroscopic images of the bare and coated scaffolds after 21 days of cell culture; and (**b**) qRT-PCR analysis to assess the expression of osteogenic markers in hMSCs and the co-culture of hMSCs and hEPCs at a 50:50 seeding ratio: PCL/HA-composite scaffolds contain a PDA@B/PDA@V surface coating consisting of two polydopamine (PDA) layers connected by a dithiol linker and entrapping BMP-2 (B) and VEGF (V) growth factors within the first and second PDA layers, respectively.

Osteogenesis of hMSCs was evaluated by staining and quantifying calcium deposition and by analysing the gene expression of osteogenic markers. Since, during osteogenesis, osteogenic cells secrete mineral deposits, high mineral accumulation is usually an indication of complete osteogenic differentiation. Thus, the mineralization degrees after 1, 21 and 28 days of cell culture were assessed employing Alizarin Red quantification for calcium deposition. Figure 7a shows that the mineralization induced by PDA@B/PDA@V-coated scaffolds in co-culture conditions is higher than for both hMSCs and hEPCs in monoculture conditions. In particular, an approximately 1.2-fold increase in Alizarin Red is observed after 21 and 28 days of cell culture in co-culture conditions as compared to hMSCs monoculture. While macroscopic imaging of the scaffolds does not allow for appreciation of the differences in the degree of mineralization for mono- and co-culture conditions, an enhanced degree of mineralization can be observed for PDA@B/PDA@V-coated scaffolds as compared to the bare ones. Next, the gene expressions of three crucial osteogenic markers (i.e., RUNX-2, ALP and OCN) were quantified in co-culture conditions for bare and PDA@B/PDA@V-coated scaffolds at 1, 3, 7 and 14 days of post-seeding. The results, which have been normalized to the gene expression of the different osteogenic markers following 1 day of hMSC cell culturing, show upregulation in co-culture conditions for the three markers (Figure 7b). Thus, while no differences in the expression of the different markers can be observed for the bare scaffolds, approximately 1.2-, 1.1- and 1.3-fold increases for RUNX-2, ALP and OCN, respectively, can be observed for hMSCs co-cultured onto the PDA@B/PDA@V-coated scaffolds as compared to hMSCs monocultured onto the same coated scaffolds. These results highlight how the PDA@B/PDA@V surface coating coupled to the co-culturing condition can promote the gene expression of three different osteogenic markers leading to earlier phenotypic commitment of hMSCs to osteogenic differentiation.

### 2.9. Angiogenesis of hEPCs in Co-Culture Conditions

**Figure 8:**
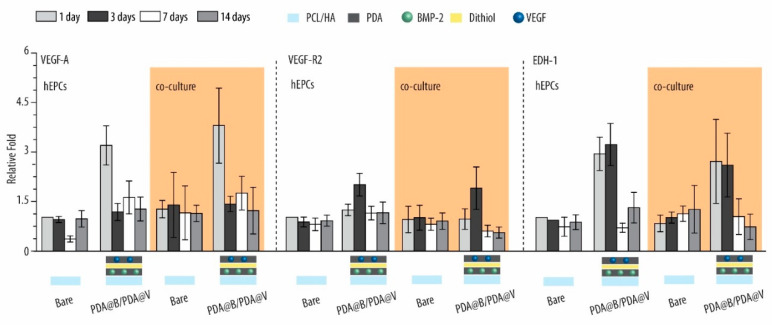
qRT-PCR analysis to assess the expression of angiogenic markers in human endothelial progenitor cells (hEPCs) and the co-culture of hEPCs and human mesenchymal stem cells at a 50:50 seeding ratio: PCL/HA-composite scaffolds contain a PDA@B/PDA@V surface coating consisting of two polydopamine (PDA) layers connected by a dithiol linker and entrapping BMP-2 (B) and VEGF (V) growth factors within the first and second PDA layers, respectively.

The gene expression of three prominent angiogenic markers (i.e., VEGF-A, VEGF-R2 and EDH-1) was also evaluated in co-culture conditions and compared to the expression in hEPC monocultures. Figure 8 shows how, while no difference in co-culture conditions could be observed for VEGF-R2 and EDH-1 as compared to hEPC monocultures, the results were different for VEGF-A. In particular, approximately 1.25-fold increase in VEGF-A expression could be detected for the PDA@B/PDA@V-coated scaffolds in co-culture conditions as compared to hEPCs monocultures. Despite the slightly higher upregulation, such a result highlights the ability of the PDA@B/PDA@V coatings together with the co-culturing conditions to stimulate angiogenesis. These results are in agreement with previous reports showing augmented VEGF expression and protein secretion for RGD-functionalized scaffolds when co-culturing hMSCs and HUVECs [52].

## 3. Materials and Methods

### 3.1. Materials

PCL (Mw = 80 kDa, T*_m_* = 59–64 °C), HA-NPs (<200 nm), ethyl acetate (EA), ethyl lactate (EL), 1,9-nonanedithiol, tris(hydroxymethyl)aminomethane (TRIS), phosphate buffered saline (PBS), sodium chloride (NaCl), DA, alizarin red S, cetylpyridium chloride and sodium dihydrogen phosphate (NaH_2_PO_4_) were obtained from Sigma-Aldrich (Saint Louis, MO, USA). RNeasy Mini Kit was purchased from Qiagen. Recombinant BMP-2, VEGF, BMP-2 human ELISA Kit, VEGF human ELISA Kit, SuperScript™ III One-Step RT-PCR System with Platinum™ Taq High Fidelity DNA Polymerase and mammalian protein extraction reagent (M-PER^®^) were purchased from ThermoFisher Scientific (Waltham, MA, USA). SensoLyte^®^ pNPP Alkaline Phosphatase Assay Kit was purchased from Anaspec (Fremont, CA, USA).

RUNX-2 (unique assay ID: qHsaCID0006726), ALP (unique assay ID: qHsaCED0045991), OCN (unique assay ID: qHsaCED0038437), VEGF-A (unique assay ID: qHsaCED0043454), EDH-1 (unique assay ID: qHsaCEP0058560), VEGF-R2 (unique assay ID: qHsaCID0006310) and β-actin (ACTB, unique assay ID: qHsaCED0036269) primers were purchased from Bio-Rad (Bio-Rad, Copenhagen, Denmark).

Two types of buffer solutions were used throughout all experiments: TRIS#1 buffer composed by 10 mM TRIS at pH 7.4 and TRIS#2 buffer composed by 10 mM TRIS and 150 mM NaCl at pH 7.4. The buffer solutions were prepared with ultrapure water (Milli-Q (MQ) gradient A 10 system, resistance 18 MV cm, TOC < 4 ppb, EMDMillipore, USA).

### 3.2. Cell Culture

Triton X-100, bovine serum albumin (BSA), phalloidin-TRITC, paraformaldehyde (PFA), ethanol (EtOH), penicillin/streptomycin, foetal bovine serum (FBS), trypsin and PrestoBlue^®^ cell viability reagent kit were obtained from Sigma-Aldrich (Saint Louis, MO, USA). DAPI, secondary antibody Alexa Fluor 488 anti-rabbit and primary antibody rabbit anti-PECAM-1 were purchased from ThermoFisher Scientific (Waltham, MA, USA). The hMSCs were purchased from American Type Culture Collection (ATCC, Manassas, VA, USA), while the hEPCs were obtained from Celprogen (Torrance, CA, USA). Mesenchymal stem cell growth medium BulletKit™ (MSCGM™), mesenchymal stem cell basal medium (MSCBM™) and MSCGM™ SingleQuots Supplement Kit were purchased from Lonza (Basel, Switzerland), and the Endothelial Cell Medium Kit (ECMK) was obtained from Innoprot (Bizkaia, Spain).

The hMSCs were cultured in MSCGM™ BulletKit™ consisting of MSCBM™ basal media supplemented with MSCGM™ SingleQuots Supplement Kit, 10% FBS and 1% penicillin/streptomycin solution. hEPCs were grown in ECM consisting of endothelial basal medium, 1% endothelial cell growth supplement, 5% FBS and 1% penicillin/streptomycin solution. Upon reaching approximately 80% confluence, the hMSCs and hEPCs were detached from the culture flask by adding trypsin (approximately 5 mL for a 75 cm^2^ flask) for 5 min. The suspension of hMSCs and hEPCs was then spun down at 200× *g* for 5 min and resuspended in new culture medium at a concentration of 20 × 10^3^ cells in 15 µL, which will be subsequently added onto each scaffold sample. Only hMSC and hEPC passages between 2 and 5 were used.

### 3.3. Fabrication of the Scaffolds

#### 3.3.1. Fabrication of PCL/HA-Based Scaffolds

The scaffolds were prepared as previously described [15,16]. Briefly, 0.06 g of HA-NPs was added to 2 mL of EL and then magnetically stirred for 30 min followed by sonication for another 30 min. A homogeneous suspension was obtained. Under continuous magnetic stirring, the temperature was increased to 60 °C followed by the addition of 0.6 g of PCL. The mixture was kept at 60 °C while continuously stirring for approximately 12 h until PCL was completely dissolved. The obtained white suspension was then cooled down by placing it for 3 h in the freezer at −20 °C, thus inducing gelation. Following that, EL was extracted from the gel by adding cold MQ, sonicating the mixture (15 min) and filtering out the resulting precipitate. EL extraction was conducted for at least three times to yield approximately 0.3 g of a PCL/HA-based precipitate that was subsequently dehydrated in EtOH. To do so, cold EtOH was added to the precipitate followed by sonication (15 min). The procedure of EL extraction was repeated two more times upon filtering out the precipitate. Finally, the PCL/HA-based precipitate was dried at room temperature and ambient pressure.

Next, 2-mm-thick disc-shaped tablets of 13 mm in diameter containing 0.3 g of the PCL/HA-based precipitate were prepared employing a hydraulic press at 3.6 MPa. For the supercritical (sc) CO_2_ foaming process, a high-pressure vessel of 100 mL was employed. The PCL/HA tablets were placed on top of a metallic support. EA (100 mL) was placed at the bottom of the vessel, and a magnetic stirrer was used to enhance the mixing of CO_2_, thus reducing the equilibrium time. Following that, the temperature was elevated to 45 °C, followed by the addition of liquid CO_2_ to the vessel, making use of a high-pressure pump (500D Syringe Pump, Teledyne ISCO, NE, USA). The working pressure was 20 MPa [53,54,55]. After 12 h, the pressure was damped under control to ambient conditions to induce foaming. A two-step depressurization profile was conducted to design the pore size distribution. On the first step, the pressure was slowly released from 20 to 7 MPa over a period of 60 min following re-equilibration of the temperature to 45 °C. On the second depressurization step, the pressure was fast released to ambient conditions over a period of only 5 min. Such a two-step depressurization approach was chosen to render a bimodal architecture of large and small pores. The first slow depressurization step reduces pore nucleation phenomena while promotes pore growth, thus rendering large pores [31]. Contrarily, the second fast depressurization step induces a fast pore nucleation rate and creates small pores within the scaffold. Finally, the tablets were snap-frozen in liquid nitrogen and cut in pieces (approximately 0.5 cm × 0.5 cm × 0.5 cm in size) by using a razor blade.

#### 3.3.2. Characterization of the PCL/HA-Based Scaffolds

The surface morphology of the scaffolds was analysed by SEM using a FEI Quanta 200 ESEM FEG (FEI-Company, Fremont, CA, USA). The macro- and microporosity were evaluated by cross-sectioning them with a razor blade followed by gold-sputtering (10 nm thickness), employing a Q150T ES Turbo-Pumped Sputter (Quorum Technologies, Lewes, UK). The working distance and potential employed were 10 mm and 5 kV, respectively.

The porosity of the samples was calculated by the following Equation (4):

(4) $$\mathrm{Porosity}\;\left(\%\right)=\left[1-\left(\frac{\rho_{\mathrm{scaffold}}}{\rho_{\mathrm{material}}}\right)\right]\times 100$$

where ρ_scaffold_ is the density of the sample which was obtained from mass and volume measurements using a high-accuracy balance (X105 Dual range, Mettler Toledo, Denmark) and a high-precision calliper, respectively, and ρ_material_ is the density of the bulk material obtained from the theoretical densities of both composite components, PCL and HA. Three different measurements were conducted independently, and the pore size distribution of the scaffolds was evaluated by image (Image J^®^) analysis. At least three different samples were evaluated, and 100 pores were analysed for each sample.

#### 3.3.3. Surface Functionalization

The coating of the scaffold samples was conducted following a previously reported protocol [16]. Briefly, the samples were weighed with a high-accuracy balance, placed in individual vials and incubated for 2 h in a DA solution (300 µL of 40 mg mL^−1^ DA in TRIS#1 for a 50-mg scaffold sample). When needed by experiment design, BMP-2 (1 µg mL^−1^) was incorporated into the DA solution. The scaffold samples were then rinsed 3× in TRIS#2 to remove PDA excess and the unreacted DA. To adsorb a second PDA layer, the samples were immersed in a 1, 9-nonanedithiol solution (1 mg mL^−1^ in TRIS#1) for 1 h. The samples were then rinsed 3× in TRIS#2 and incubated in a DA solution for 2 h (300 µL of a 40 mg mL^−1^ DA solution in TRIS#1 for a 50-mg scaffold sample) to create the second PDA layer. When needed by experiment design, VEGF (0.5 µg mL^−1^) was incorporated into the DA solution. The samples were then rinsed 3× in TRIS#2. Finally, the coated scaffold samples were dried under vacuum overnight.

##### TGA

Successful deposition of the different layers was evaluated by TGA employing a Q500 TGA (TA Instrument, USA). The different samples were heated from 30 to 800 °C at a rate of 10 °C min^−1^ under constant nitrogen flow of 60 mL min^−1^. The mass loss as a function of the temperature was monitored. Two samples were studied for each condition.

##### XPS

The chemical composition of the adsorbed material following each deposition step was evaluated by XPS conducted in a Thermo Scientific K-Alpha XPS system (ThermoFisher Scientific, Hvidovre, Denmark) operating with an EX06 ion source and a hemispheric analyser with 180° double focus and a 128 channel detector. Two samples were studied for each working condition.

##### ELISA

The amount of BMP-2 and VEGF entrapped within the scaffolds was determined as the difference between the total amount of GFs present in the starting and final solutions. A Human BMP-2 ELISA Kit and a VEGF Human ELISA Kit from Invitrogen (CA, USA) were used. Standard curves for both BMP-2 and VEGF were created by employing known concentrations of the two GFs. These experiments were performed in triplicate.

#### 3.3.4. BMP-2 and VEGF Release Kinetics

The in vitro release of both GFs from the scaffolds was evaluated as follows. About 50 mg (weighted precisely) of the different scaffold samples was incubated in PBS (220 µL) under constant shaking for 20 days at 37 °C. At the different time intervals (i.e., 1, 5, 10, 15 and 20 days), the supernatants were collected and replaced by fresh PBS (220 µL). The amount of GFs in the supernatant was determined by employing the aforementioned ELISA kits and the corresponding standard curves. These experiments were performed in triplicate.

### 3.4. Cell Viability, Proliferation and Adhesion of hMSCs and hEPCs

The scaffolds were first sterilized by immersion in 70% ethanol for 30 min. After being washed 3× in PBS, they were placed in 96-well plates and preincubated for 6 h in complete MSCGM medium (for hMSCs) or ECM medium (for hEPCs) at 37 °C and 5% CO_2_. The scaffolds were then transferred to a new 96-well plate, and a cell suspension (20 × 10^3^ hMSCs and 20 × 10^3^ hEPCs cells in 15 µL, respectively) was slowly and uniformly added to the scaffolds. Approximately 80–90% of the scaffolds surface was covered by the cells. The well plates were incubated (4 h at 37 °C and 5% CO_2_), and 10 µL of fresh medium was added to each scaffold every 30 min to prevent the cells from drying out. Next, 300 µL of fresh medium was added to each well and the samples were incubated for the required time period, always changing the cell medium every other day.

#### 3.4.1. Cell Viability

Cell viability was determined at various time points (1, 7, 14 and 21 days). Cells were washed 2× in PBS, and a mixture of PrestoBlue (15 µL) and complete cell media (135 µL) was added to each well. After incubation for 1 h in the dark at 37 °C and 5% CO_2_, the fluorescence intensity of the reduced resazurin compound was determined at 535/615 nm for excitation and emission wavelengths, respectively (Tecan Spark, TECAN, Männedorf, Switzerland). Cell viability was calculated as follows: % cell viability = (experimental value − negative control value)/(positive control value − negative control value) × 100. Each condition was evaluated in triplicate in at least two independent experiments.

#### 3.4.2. Cell Proliferation

The number of cells was evaluated at the different time intervals (i.e., 1, 7, 14 and 21 days) by washing the cells 2× in PBS and by adding a solution of PrestoBlue (15 µL) in cell media (135 µL) to each well. Following incubation for 1 h in the dark at 37 °C and 5% CO_2_, the fluorescence intensity of the reduced resazurin product at 615 nm (excitation wavelength of 535 nm) was read with a microplate reader (Tecan Spark, TECAN, Männedorf, Switzerland). For correlation of the absorbance values to the number of cells, standard curves were generated. Known numbers of cells were seeded, and after 24 h of incubation, the fluorescence intensity of the supernatants was evaluated at 615 nm employing an excitation wavelength of 535 nm.

#### 3.4.3. Cell Adhesion

The nuclei and actin filaments of both hMSCs and hEPCs were stained after different time points (i.e., 1, 7 and 14 days). After the incubation times, the wells were rinsed (3× in PBS) and fixated. The cells were then incubated in a 4% (*v*/*v*) PFA solution in PBS for 20 min. The cells were washed (3× in PBS) and permeabilized. For that, a 0.1% Triton X solution in PBS was employed for 15 min. The cells were next incubated for 1 h at room temperature (RT) in a 1% BSA solution in PBS in order to block the nonspecific points. Following several washes in PBS, the actin filaments were stained by incubating the cells in a phalloidin-TRITC solution (3 µg mL^−1^ in PBS) for 1 h also at RT. The nuclei staining was conducted by incubating the cells in a DAPI solution (20 µg mL^−1^) for 5 min at RT. Following several washes in PBS, the stained cells onto the different scaffold samples were visualized by CLSM using a Yokogawa CSU-W1 spinning disk confocal microscope (Nikon, Tokyo, Japan).

### 3.5. Osteogenic and Angiogenic Gene Expression by qRT-PCR

Osteogenic and angiogenic gene expression were evaluated at 1, 3, 7 and 14 days by RT-qPCR. As osteogenic markers, RUNX-2, ALP and OCN were selected, while as angiogenic markers, VEGF-A, VEGF-R2 and EDH-1 were chosen. The mRNA of both hMSCs and hEPCs was extracted and collected at different time intervals employing the RNeasy Mini Kit (Qiagen, Hilden, Germany). In particular, the concentration of total RNA was quantified by UV-vis using a Nanodrop ND-1000 spectrophotometer (NanoDrop Technologies, Montchanin, DE, USA). If RT-qPCR was not going to be immediately conducted, the samples were stored at −80 °C. The RT-qPCR of the RNA targets (0.1 µg) was conducted employing an iTaq universal SYBR Green One-Step kit (Bio-Rad, Denmark) in a Chromo4™ Detector (Bio-Rad). The primers for the amplification of specific genes of both osteoblast and angiogenic differentiation were purchased from Bio-Rad Laboratories (Bio-Rad). The amplifications were conducted following this protocol: a first heating step for 10 min at 50 °C, a second heating step for 1 min at 95 °C, 40 cycles at 95 °C for 15 s and, finally, 40 cycles at 55 °C for 30 s. The different gene expressions were normalized to the expression levels of ACTB (reference gene). The relative fold changes were correlated to 1-day gene expression of either hMSCs or hEPCs cultured onto the bare PCL/HA composite scaffolds. The fold change (*FC*) was calculated as follows:

(5) $$FC=\frac{E_{target}{}^{\Delta C_{q,target\left[PCL/HA,\;1d\right]}}}{E_{reference}{}^{\Delta C_{q,reference\left[PCL/HA,\;1d\right]}}}$$

where *Cq* corresponds to the median value for the quantification cycle of the triplicate of each sample. *E* is the efficiency amplification, and it is determined from the slope of the log-linear portion of the calibration curve, as follows:

(6) $$E=\;10^{1/slope}$$

Two independent experiments were conducted in triplicate.

### 3.6. Optimization of hMSCs and hEPCs Co-Culture Ratio

hMSCs and hEPCs were expanded separately as described in Section 3.2. Upon reaching approximately 80% confluence, the cells were detached and seeded at different ratios (i.e., hMSCs:hEPCs of 4:1, 2:1, 1:1, 1:2 and 1:4) onto 96-well plates at a seeding density of 20,000 cells per well. The cell mixture was cultured for different time periods (i.e., 1, 7, 14 and 21 days) in ECM medium at 37 °C and 5% CO_2_. The medium was replaced every second day. The optimal ratio of hMSCs–hEPCs for the co-culture conditions was identified by assessing the percentage of both cell lines at different time points and by evaluating the proliferation of the cells.

#### 3.6.1. Percentage of hMSCs and hEPCs

After the different time intervals of cell co-culture (i.e., 1, 7, 14 and 21 days), the wells were washed 3× with PBS (200 µL) and incubated with trypsin to detach the cells. The percentages of hMSCs and hEPCs were identified by flow cytometry employing a BD Accuri C6 flow cytometer (BD Biosciences) and by monitoring the forward and the side scattering. At least 3000 cells were analysed, and three independent repeats were performed.

#### 3.6.2. Cell Proliferation

The number of cells was evaluated at the different time intervals (i.e., 1, 7, 14 and 21 days) by employing the PrestoBlue assay as described in Section 3.4.1.

#### 3.6.3. Alkaline Phosphatase Activity

Following incubation for 30 min, the cellular ALP activity was investigated via a p-nitrophenol phosphate (pNPP) assay. In particular, at each time interval, the medium of each well was carefully removed and the cells were washed 3× in PBS and lysed with 200 µL of M-PER^®^ for 10 min. The cell lysates were collected in Eppendorf tubes and stored at −80 °C. Fifty microlitres of each lysate were incubated with 50 µL of pNPP for 30 min at RT using a SensoLyte^®^ pNPP Alkaline Phosphatase Assay Kit (AnaSpec, Inc., Fremont, CA, USA). The p-nitrophenol reaction product was monitored by UV-vis at 405 nm using the multiplate reader. The quantity of p-nitrophenol produced by the cells was measured by comparing the absorbance readings against a calibration curve prepared using standard p-nitrophenol solutions. The ALP activity was represented as pg of p-nitrophenol, normalized to the number of cells obtained by the cell proliferation assay and divided by the incubation time (30 min).

### 3.7. Characterization of Co-Cultures of hMSCs and hEPCs

The cells were seeded at a 50:50 hMSCs–hEPCs ratio. At different time points (i.e., 1, 7 and 14 days of co-culture), the cells were stained for nuclei, actin filaments and endothelial PECAM-1. Briefly, at each time point, the scaffold samples were washed 3× with PBS and fixated with a 4% (*v*/*v*) PFA solution in PBS for 20 min at RT. Following several washes in PBS, the permeabilization process was performed using a 0.1% Triton X solution in PBS for 15 min. Next, the cells were 3× washed in PBS and incubated with a blocking solution of 0.1% BSA in PBS for 1 h at RT. Following several washes in PBS, the scaffold samples were incubated with the primary antibody rabbit anti-PECAM-1 (1:100 in 1% BSA in PBS) for 1 h. The samples were rinsed 3× in PBS, followed by incubation with a secondary antibody Alexa Fluor 488 anti-rabbit (1:1000 in PBS) and a phalloidin-TRITC solution (3 µg mL^−1^ in PBS) for 1 h also at RT. For nuclei staining, the cells were incubated in a DAPI solution (20 µg mL^−1^ in PBS) for 5 min at RT. The stained cells onto the different scaffold samples were visualized using the CLSM.

### 3.8. Alizarin Red Quantification in Co-Culture Conditions

Calcification of the cellular matrix of the hMSC–hEPC co-culture was determined at 1, 21 and 28 days using Alizarin Red staining (ARS). After culturing time, cells were 3× washed in PBS and fixed with 4% (*v*/*v*) PFA for 20 min at RT. Then, samples were 3× washed in MQ water and immersed in 300 µL of an ARS solution (40 mM in MQ water, pH 4.2) for 20 min at RT under continuous shaking. Next, sequential washes in MQ water were performed in order to remove excess stain. For cell quantification, the stain was eluted with a cetylpyridinium chloride buffer (10% (*w*/*v*) in 10 mM NaH_2_PO_4_, pH 7.0) by incubation for 30 min at RT. Finally, 100 µL of the extracted stain was transferred to a 96-well plate and the absorbance at 570 nm was measured using the microplate reader.

### 3.9. Osteogenic and Angiogenic Gene Expression in Co-Culture Conditions by qRT-PCR

Osteogenic and angiogenic gene expression in co-culture conditions were evaluated at 1, 3, 7 and 14 days of co-culture conditions by qRT-PCR, as previously described in Section 3.5. Two independent experiments were conducted in triplicate.

### 3.10. Statistical Analysis

All data were analysed using one-way ANOVA with a confidence level of 95% (α = 0.05) followed by Tukey‘s multiple comparison post hoc test (*p ≤ 0.05, **p ≤ 0.01, *** *p* ≤ 0.001 and **** *p* ≤ 0.0001) in a GraphPad Prism 7 software

## 4. Conclusions

By making use of a super critical CO_2_ foaming procedure, PCL/HA-composite scaffolds with a highly interconnected network of pores and a bimodal pore size distribution mimicking the extracellular matrix of bone tissue were fabricated. The as-prepared scaffolds were subsequently coated by two independent PDA layers loaded with different GFs. Although the immobilization of GFs within PDA coatings has previously been shown, entrapment of two different GFs within separated and independent PDA layers is reported here for the first time. BMP-2 and VEGF were the chosen GFs and were incorporated within the two different PDA layers. While BMP-2 is a well-known, FDA-approved osteogenic GF, VEGF plays a central role in angiogenesis, thus promoting vascularized bone regeneration. VEGF is entrapped within the second and more exposed PDA layer, while BMP-2 is immobilized in the first and more hindered one. Thus, this design promotes a fast VEGF release together with a slow and more sustained release of BMP-2. Therefore, such a release profile matches the needs for BTE applications since, during the natural process of bone healing, VEGF secretion mainly occurs at an early healing stage while BMP-2 release takes place during the entire process. The potential of this multilayered scaffolds was evaluated in monocultures of hMSCs and hEPCs showing pronounced upregulation of three different osteogenic (i.e., RUNX-2, ALP and OCN) and angiogenic (i.e., VEGF-A, VEGF-R2 and EDH-1) markers. These results highlight the ability of this multilayered coating in stimulating both angiogenesis and osteogenesis. The ability of the coated scaffolds in regulating crosstalk among bone- and vessel-forming cells was evaluated through a co-culture system of hMSCs and hEPCs. Importantly, evaluation of angiogenic and osteogenic gene expression demonstrated how the developed surface coatings coupled to the co-culture conditions resulted in a synergistic effect on both osteogenic and angiogenic events.

Despite the potential of biomaterials-based scaffolds reported by us [16] and others [56], the translation of in vitro BTE into a clinical reality is a tremendously slow process resulting from the intrinsic complexity of biological tissues. There is a long list of required features that explain why an off-the-shelf scaffold for BTE applications has not been developed yet. Some of these demanded features have been described in this work. For example, the need for an interconnected network of pores allowing for cell migration, bioinductive coatings for cells to proliferate and secrete GFs or the ability to stimulate osteogenesis and angiogenesis. However, other important requirements include a morphological structure resembling the injured/diseased tissue to be replaced and the ability of such a construct to integrate without rejection. Additionally, selective matrix absorption should not result in the deposition of toxic by-products that may negatively influence the mechanism of action of surrounding cells and tissues.

## Abbreviations

PDA	Polydopamine
BTE	Bone tissue engineering
3D	Three-dimensional
HA	Hydroxyapatite
PCL	Poly(ɛ-caprolactone)
HA-NPs	Hydroxyapatite nanoparticles
ECM	Extracellular matrix
GF	Growth factor
BMP-2	Bone morphogenetic protein-2
VEGF	Vascular endothelial growth factor
EPC	Endothelial progenitor cells
DA	Dopamine
hMSCs	Human mesenchymal stem cells
hEPCs	Huma endothelial progenitor cells
TGA	Thermogravimetric analysis
PDA@B	PDA-containing BMP-2 coated scaffold
PDA@V	PDA-containing VEGF coated scaffold
PDA@B+V	BMP-2 and VEGF growth factors within the same layer coated scaffold
PDA@B/PDA@V	BMP-2 and VEGF growth factors within two different layers separated by a dithiol layer coated scaffold
MPDA	Amount of PDA deposited
XPS	X-ray photoelectron spectroscopy
CLSM	Confocal laser scanning electron microscopy
RUNX2	Runt-related transcription factor 2
OCN	Osteocalcin
VEGF-A	Vascular endothelial growth factor A
EDH-1	Endothelin 1
VEGF-R2	Kinase insert domain receptor
ACTB	β-actin
BSA	Bovine serum albumin
PFA	Paraformaldehyde
DAPI	4′,6-diamidino-2-phenylindole
ELISA	Enzyme-linked Immunosorbent Assay
ALP	Alkaline Phosphatase
pNPP	p-Nitrophenol phosphate
PECAM-A	Platelet endothelial cell adhesion molecule A
ARS	Alizarin Red staining
TRIS	1,9-nonanedithiol, tris(hydroxymethyl)aminomethane
PBS	Phosphate buffered saline
M-PER^®^	Mammalian protein extraction reagent
NaCl	Sodium chloride
NaH_2_PO_4_	Sodium dihydrogen phosphate
